# Supplementary material for: Cultural differences in healthcare: An investigation using cognitive-affective mapping
Source: Glob Ment Health (Camb). 2025 Dec 26;13:e31. doi: 10.1017/gmh.2025.10126 (PMC12951343; doi:10.1017/gmh.2025.10126)
Supplement: Buschmeyer et al. supplementary material [file S205442512510126Xsup001.pdf]

**Supplementary Material**

Cultural Differences in Healthcare:
An Investigation Using Cognitive-Affective Mapping

Contents

Supplementary Table S1 Category system - supporting factors for refugees in Germany in the context of healthcare (179 concepts). 2

Supplementary Table S2 Category system – hindering factors of refugees in Germany in the context of healthcare (141 concepts). 5

Supplementary Table S3 Category system – descriptive concepts used by refugees in Germany in the context of healthcare (35 concepts). 9

Supplementary Table S1

Category system - supporting factors for refugees in Germany in the context of healthcare (179 concepts).

Main category	Subcategory	Dimension	Coding Rule	Concepts mentioned in CAMs
Resources [a] (R)<br>Resources are factors that individuals can use to deal with stress and reduce stressors specific to their life situation	Available Resources [a] (R-AR)<br>All internal factors that are available to the participants to cope with stressful situations	• Personal capital (e.g. education, personality, language skills)<br>• Useful tools (e.g. mobile phone)<br>• Work	Compared to "R-CS", this concept refers to inner resources, which are already developed, rather than strategies, which must be actively applied externally to have an effect.	Personal capital: English knowledge; freedom; independence; learning German; own initiative; own knowledge research; patience; projects [to improve German]; sensitise [knowledge of supporting possibilities]; you can plan [in Germany]; *approach people; self-education;*<br>Useful tools: Internet; mobile phone;<br>Work: good work; hard working; many mini jobs currently[2]; money; suitable work; work;
	Coping strategies [a] (R-CS)<br>Active strategies for everyday and indirect disease management	• Creative-artistic activity (e.g. painting, music)<br>• Exercise/ Sport (e.g. walking, dancing)<br>• Food (no drinks)<br>• Media consumption (e.g. books, movies)<br>• Relaxation<br>• Religious/ spiritual activities<br>• (Visiting) Certain places (e.g. nature, to be at home)<br>• Voluntary work (e.g. working in a cultural centre)<br>• Other activities (e.g. stay in bed)	Compared to "R-AR", this concept focuses on actively applied externally strategies, rather than already developed inner skills.	Creative-artistic activity: drawing; listening to music; make music yourself; music; singing; working in a bicycle repair shop;<br>Exercise/ Sport: bicycle path; gymnastics, jogging; movement; sport[8]; swimming; walking[5]; walking with family; yoga[2];<br>Food: delicious & healthy food; eat healthily; eating sweets; food; more healthy food; products show ingredients;<br>Media consumption: reading; watch a good movie; watch comedy;<br>Relaxation: bathtub; calm myself down; keep mind free; massage; relaxation; releasing stress; resting; silence; smoking weed;<br>Religious/ spiritual activities: book by Grigori Grabovoi; course in parapsychology; esotericism; listening Christian music; praying; reiki; work with energies;<br>(Visiting) certain places: forests; going outside; Gundelfingen; Language café in city library; nature; outside; parks; social garden; to be alone; town hall [offers all

Main category	Subcategory	Dimension	Coding Rule	Concepts mentioned in CAMs
		• Effects of successful coping strategies (e.g. feeling better)		relevant information]; trips in Germany; Ukrainian cultural centre; *being alone; spending time alone;*<br>Voluntary work: helping at the cultural centre; helping other people;<br>Other activities: crying in bedroom; smoking; stay in bed; talking about women's rights;<br>Effects: distraction; less anxiety; more energy; relief;
	Social support [a] (R-SS)<br>Support from private individuals to deal with the experience of being a foreigner in the host country and in the care system	• Family & Partnership<br>• Friends & acquaintances<br>• Form of support (e.g. reading letter)	Compared to "T-P", this concept focuses on social support from private individuals, rather than direct healthcare treatment by professionals.	Family & Partnership: ask my family; be with family; call mum; family; family helps; family support; husband helps; parents; relationship with partner; relatives; talking with mother; wife;<br>Friends & acquaintances: already know people; ask friends for experiences; doctors in the circle of acquaintances; easy-going & subliminal; friends[2]; friends help; German friends; help from acquaintances; meet many people; meet new people; meeting friends; meeting good friends; nice people[2]; people helping us[2]; talk to friends on the phone*; help from friends; talk to friends;*<br>Form of support: help reading letter; information [e.g. to see doctor without health insurance]; support; support in everyday life; support with [translation]; talking (with people/ about it); Ukrainian language charts [info written by Ukrainian people with longer experience with living in Germany]; *commitment;*
Explicit treatment of healthcare needs [b] (T)<br>Usage of professional or self-administered healthcare	Professional treatment (T-P)<br>Context factors of direct disease management strategies that require healthcare professionals to implement	• Professional field (e.g. doctor)<br>• Treatment (e.g. psychotherapy)<br>• Features of a good treatment (e.g. correct diagnosis)<br>• Positive doctor-patient-interaction	Compared to "R-CS" and "T-S" this concept focuses on direct usage of healthcare offered by professionals, rather than indirect coping strategies or self-administered healthcare.	Professional field: (seeing) doctor[14]; general practitioner; doctor Germany; physiotherapist; psychiatrist; (contact) social worker[6]; specialist doctor[3]; *general practitioner; nursing staff;*<br>Treatment: examination; medical treatment[2]; medicine/medication[11]; psychotherapy[7]; stationing; surgery[3];<br>Features of a good treatment: correct diagnosis; good qualified; higher quality Germany; prevent diseases;

Main category	Subcategory	Dimension	Coding Rule	Concepts mentioned in CAMs
		(e.g. friendly doctor)<br>• Helpful framework conditions (e.g. health insurance)<br>• Places (e.g. hospital)<br>• Action (e.g. going to hospital)<br>• Other		properly checking; prophylactic offers; right medication; understanding the diagnosis; *human factor;*<br>Positive doctor-patient-interaction: some [professionals] friendly; Romanian caregiver good; speaking easy English;<br>Helpful framework conditions: appointment after 1 day; basic knowledge about healthcare system; better medicine; English skills in the health system; health insurance[6]; instructions in various languages; insurance system; many doctors English knowledge; money from health insurance; no long waiting time; translator (person); where to get help at night? [knowledge healthcare system];<br>Places: hospital; pharmacy[6]; *ambulance; treatment in practice;*<br>Action: calling the GP; going hospital; make an appointment[4]; prepare visit; seeking help;<br>Other: healthcare in Ukraine;
	Self-administered treatment (T-S)<br>Strategies for direct disease management, implemented by themselves	• (e.g. sleeping, drinking water)	Compared to "R-CS" and "T-P" this concept focuses on direct usage of healthcare implemented by themselves, rather than indirect strategies or healthcare offers by professionals.	> 9 hours [of sleep]; self-help; self-medication; 30 years no medication; drinking sleep tea; drinking water[4]; household remedies; healing by yourself; sleeping[2]; symptoms disappear on their own; *self-diagnosis difficult.*

Note. Concepts in italics indicate neutral valence; they were also categorised in the Supplementary Table S2 coding guideline. Information in parentheses indicates participants' comments provided to understand the concept. Superscript numbers indicate the number of participants who contributed to the respective concept if there was more than one participant.

[a] Deductively developed categories based on the category system of Nowak, 2022.
[b] Inductively developed categories.

Supplementary Table S2
Category system – hindering factors of refugees in Germany in the context of healthcare (141 concepts).

Main category	Subcategory	Dimension	Coding Rule	Concepts mentioned in CAMs
Access to healthcare [a] (AC)<br>Possibility to make use of general healthcare in Germany	Generic barriers [a] (AC-GB)<br>Barrier can affect any patient	• Doctors' shortage<br>• Negative professional-patient-interaction<br>• Costs<br>• Other	Compared to “LC-RS” exclusively focus on direct barriers regarding access to healthcare; barriers are not related to migration or refugee identity.	Doctors' shortage: difficult getting appointment[3]; few appointments; find a good doctor; little treatment time[3]; long waiting time[9]; no appointments; no new patient admission; not enough doctors; poor availability by phone; waiting for an appointment[2];<br>Negative professional-patient-interaction: incompetent medical staff; lack of treatment flexibility; main problem: no communication [nurse]; no information medication; not qualified doctor; quality of help; shouted at patients; sometimes rude; wrong diagnosis; wrong ward?; *other patients sedated?;*<br>Costs: additional costs; dentures too expensive; own contribution[2]; expensive[3]; expensive seeing doctor; financial limit; financially not possible; limitations [demand physiotherapy per quarter]; *partly cost;*<br>Other: no confidence in supply; no trust in care; some [medication] with prescription;
	Migration-specific barriers [a] (AC-MS)<br>Barrier can also occur among other migrant populations like international students or international workers	• Language-barrier-related<br>• Insufficient knowledge of German healthcare system	Compared to “LC-RS” exclusively focus on direct barriers regarding access to healthcare; barriers are not related to refugee identity.	Language-barrier-related: doctor consultation by phone; emergency phone [not] various languages; fear appointments by phone; language barrier[9]; learning German difficult; no interpreter; no psychotherapy in native language; not everyone English skills; not native German [comprehension difficulties when professionals speaking fast]; not understanding letter; people not understanding; people not knowing English; *difficult for old people; old people dialect;*<br>Insufficient knowledge: cluelessness; differences unclear; German healthcare system complicated; ignorance of the healthcare system; lack of overview; no information healthsystem[3]; no unified platform [healthcare services];

Main category	Subcategory	Dimension	Coding Rule	Concepts mentioned in CAMs
				refugees don't know; where is the doctor?; which doctor is better?; which therapy?;
	Refugee-specific barriers [a] (AC-RS)<br>Barrier only occurring due to the specific legal situation of refugees	• No health insurance-related<br>• Bureaucracy<br>• Other	Compared to “LC-RS” exclusively focus on direct barriers regarding access to healthcare; no barrier to other migrations.	No health-insurance-related: can´t get everything [what is necessary to become better]; no health insurance[3]; no treatment hospital; without insurance;<br>Bureaucracy: always proof necessary; bureaucracy[9]; many letters; migration background [overload bureaucracy]; *contacting state office[2]; sick note; state office calls hospital; no printer at home; referral;*<br>Other: unclear responsibilities;
Living conditions in Germany [b]<br>(LC)	Residence status [b] (LC-RS)		Compared to “AH”-categories focus on legal barriers independent of access to healthcare.<br><br>Compared to other “LC“-categories focus on long-term (stay) perspective, rather than immediate consequences.	diploma not recognized; fear of court; recognition of medical diploma;
	Satisfaction with accommodation [c] (LC-SA)		Compared to “LC-RS” exclusively focus on immediate accommodation-related factors, rather than long-term perspectives.	little bed camp; loud neighbour; *apartment; poor infrastructural connection;*
	Social participation [c] (LC-SP)		Compared to “PC-G” focus on current social participation and support by other people than family or partner, rather than challenges due to the relation to family or partner.	lack of social support; no contact art scene; no contact with Germans; no friends; no help; not German language [can´t talk to people]; sometimes lonely; *being alone; human factor; no German friends; spending time alone;*

Main category	Subcategory	Dimension	Coding Rule	Concepts mentioned in CAMs
	Economic participation [c] (LC-EP)		Compared to "LC-RS" exclusively focus on current economic situation, rather than long-term perspectives.	much work; no work permit[2]; no money; no work[3]; stress at work; unemployed;
Psychological challenges [c] (PC)<br>Conditions, which negatively influences the psychological well-being	Generic psychological challenges (PC-G)<br>Can affect any patient	• Family/Partner-related<br>• Emotions<br>• Maladaptive Coping Strategies<br>• Sleep difficulties<br>• Other	Psychological challenges are not related to migration or refugee identity.	Family/ partner-related: child sick; concerns about husband; conflict in relationship; dependent on partner; help myself/family; husband needs care; husband sad; (mis)treatment of husband; no partner communication; others [e.g. relatives, friends] not good; parents argue among themselves; parents in Germany; separation from partner;<br>Emotions: being lazy; depression; emotionless; feeling unwell; frustration; indifference; no desire to work; not feeling good[2]; sad; stress; *feel weak;*<br>Maladaptive coping strategies: exaggerated performance work; excessive demands[2]; stay home [all day]; unhealthy coping;<br>Sleep difficulties: < 7 hours [of sleep]; sleeping difficulties[3]; tired at school; very tired;<br>Other: being at home; being ill; forgetting many things; headaches; lack of resources; little patience; strong headache; no time;
	Migration-specific psychological challenges (PC-MS)<br>Can also occur among other migrant populations like international students or international workers	• Cultural differences<br>• Physical distance to home country	Psychological challenges are not related to refugee identity.	Cultural differences: cultural differences[3]; how does family court work?; new culture; no family communication [due to Afghan culture]; pride of husband;<br>Physical distance to home country: homesickness[2]; miss friends;
	Refugee-specific psychological challenges (PC-RS)		Psychological challenges are not related to migration identity.	discrimination; growing up in war; loss of status; not doing anything [due to no work/ work permission][2];

Main category	Subcategory	Dimension	Coding Rule	Concepts mentioned in CAMs
	Only occurring due to the specific experiences and situation of refugees			relatives in Syria [difficult living conditions]; traumas[2]; *only learn German;*
Other [c]<br>No categorisable terms				if it doesn't help; not always possible; not think for oneself [context: translation via mobile phone]; self-help; somebody disturbing me; urgent help.

Note. Concepts in italics indicate neutral valence; they were also categorised in the Supplementary Table S1 coding guideline. Information in parentheses indicates participants' comments provided to understand the concept. Superscript numbers indicate the number of participants who contributed to the respective concept if there was more than one participant.

[a] Deductively developed categories based on the taxonomy of Bozorgmehr & Gold (2023).
[b] Deductively developed categories according to Nutsch & Bozorgmehr (2020).
[c] Inductively developed categories.

Supplementary Table S3

Category system – descriptive concepts used by refugees in Germany in the context of healthcare (35 concepts).

Main category	Subcategory	Dimension	Coding Rule	Concepts mentioned in CAMs
Neutral			All concepts which are descriptive, used to structure the CAM or cannot be categorised in the regular coding system.	*Diagnosis in Ukraine; Difficulties[4]; First medical consultation; further difficulties; Help; helpful[8]; keine Verbindung[2]; Medical aids; mental health[2]; Mental health difficulties; mental help; Mental symptoms; mentally; Physical difficulty; physical health[2]; Physical help; physically; Physically ill; psychic; Somatic symptoms; when I´m sick*

Note. Superscript numbers indicate the number of participants who contributed to the respective concept if there was more than one participant.
